# Hon. Martin I. Townsend

**Published:** 1903-04

**Authors:** 


Hon. Martin I. Townsend, of Troy, died at his home March 9, 1903, aged 93 years. He had been in public life for 70 years. He was the senior regent of the University of the State of New York, having served since 1873. He was one of the most successful lawyers of his time, and a man of distinguished ability.

